# Neisseria meningitidis Antimicrobial Resistance in Italy, 2006 to 2016

**DOI:** 10.1128/AAC.00207-18

**Published:** 2018-08-27

**Authors:** Paola Vacca, Cecilia Fazio, Arianna Neri, Luigina Ambrosio, Annapina Palmieri, Paola Stefanelli

**Affiliations:** a Department of Infectious Diseases, Istituto Superiore di Sanità, Rome, Italy

**Keywords:** antimicrobial resistance, Neisseria meningitidis, *penA* gene, Pen^i^, penicillin-binding protein 2

## Abstract

The aim of this study was to evaluate the antimicrobial susceptibilities of 866 Neisseria meningitidis invasive strains during 11 years of surveillance in Italy. Two and six strains were resistant to ciprofloxacin and rifampin, respectively. Forty-five percent were penicillin intermediate, associated with hypervirulent serogroup C clonal complex 11. All of the strains were susceptible to cephalosporins.

## Abstract (precis)

The aim of this study was to evaluate the antimicrobial susceptibilities of 866 Neisseria meningitidis invasive strains during 11 years of surveillance in Italy. Two and six strains were resistant to ciprofloxacin and rifampin, respectively.

## TEXT

Invasive meningococcal disease (IMD) is a serious and rapidly progressive illness; third-generation cephalosporins or penicillin G are usually used for the treatment of patients with invasive diseases (1, 2). Ciprofloxacin or rifampin is recommended for chemoprophylaxis of close contacts of the case (2).

Although antimicrobial resistance in Neisseria meningitidis strains is rare (3), reduced susceptibility to third-generation cephalosporins has recently been reported (4). Moreover, meningococci with reduced susceptibility to penicillin G (penicillin intermediate [Pen^i^]) have been described (3, 5–7). The Pen^i^ phenotype is mainly due to the presence of five amino acid substitutions (F504L, A510V, I515V, G541N, and I566V) in the transpeptidase region of the penicillin-binding protein 2 (PBP2), encoded by the *penA* gene (8–10).

This study was conducted to evaluate the antimicrobial susceptibilities of 866 meningococcal invasive strains isolated from 2006 to 2016 in Italy. Genotyping and determination of the *penA* gene of Pen^i^ strains from 2014 to 2016 were also performed.

Clinical data, strains, and/or clinical samples of each IMD case are collected throughout the country and sent to the National Reference Laboratory (NRL) at the Istituto Superiore di Sanità (ISS), within the activities of the National Surveillance System.

Strains were cultured on Thayer-Martin agar plates with IsoVitaleX 2% (Oxoid, Ltd.) in 5% CO_2_ atmosphere at 37°C. Serogroup by slide agglutination with commercial antisera (Remel Europe, Ltd., UK) or by multiplex PCR was determined (11).

Antimicrobial susceptibility testing for ceftriaxone, cefotaxime, ciprofloxacin, penicillin G, and rifampin was performed using Etest (bioMérieux, Sweden) and MIC test strip methods (Liofilchem Diagnostici, Italy) interpreted according to European Committee Antimicrobial Susceptibility Testing (EUCAST; v. 7.1, 2017-03-10) (12). In this study, MIC values ranging from 0.094 to 0.25 μg/ml define the Pen^i^ phenotype.

DNA was extracted using QIAamp DNA minikit (Qiagen, Hilden, Germany) for whole-genome sequencing (WGS) (13). Genomes were uploaded and analyzed on the Neisseria PubMLST database (http://pubmlst.org/neisseria/). Multilocus sequence typing (MLST), porin A (PorA) and ferric enterobactin transport protein A (FetA) typing, and the *penA* allele were identified as described in the database. The genotypic formula is identified as follows: capsular group: *porA* (P1); variable region 1 (VR1), VR2 : FetA VR: sequence type (ST) clonal complex (CC).

Statistical analysis was performed by the χ^2^ test. A *P* value of <0.05 was considered to be statistically significant.

From 1 January 2006 to 31 December 2016, a total of 1,188 samples from IMD cases were received at the NRL, of which 866 samples (866/1,188 [73%]) were culture positive. As shown in Table 1, all meningococci were susceptible to ceftriaxone (866/866 [100%]) and to cefotaxime (227/227 [100%]). Except for two samples, meningococci were susceptible to ciprofloxacin (864/866 [99.7%]). Those resistant (MICs, 0.064 μg/ml and 0.19 μg/ml, respectively) were from serogroups A and C, collected from unvaccinated adults with meningitis.

**TABLE 1:** Antimicrobial and susceptibility categories identified in 866 Neisseria meningitidis strains by year, 2006 to 2016

Susceptibility category (MIC) by antimicrobial (μg/ml)^*a*^	No. of strains by yr (*n*)											
	2006 (88)	2007 (80)	2008 (101)	2009 (104)	2010 (69)	2011 (66)	2012 (62)	2013 (69)	2014 (60)	2015 (72)	2016 (95)	Total (866)
Ceftriaxone												
S ≤ 0.125	88	80	101	104	69	66	62	69	60	72	95	866
Cefotaxime												
S ≤ 0.125	NA^*b*^	NA	NA	NA	NA	NA	NA	NA	60	72	95	227
Ciprofloxacin												
S ≤ 0.03	88	80	101	103	68	66	62	69	60	72	95	864
R > 0.03	0	0	0	1	1	0	0	0	0	0	0	2
Rifampin												
S ≤ 0.25	88	79	101	101	69	66	60	69	60	72	95	860
R > 0.25	0	1	0	3	0	0	2	0	0	0	0	6
Penicillin G												
S ≤ 0.06	49	54	69	69	38	39	25	34	22	32	41	472
0.094 < I < 0.25	34	26	32	34	31	27	37	35	38	40	54	388
R > 0.25	5	0	0	1	0	0	0	0	0	0	0	6

a S, susceptible; R, resistant; I, intermediate.

b NA, not applicable; antimicrobial susceptibility testing for cefotaxime was evaluated starting from 2014.

Six strains were rifampin resistant (6/866 [0.7%]), with 3 strains from serogroups B, C, and NG, with MIC values ranging from 0.38 μg/ml to 2 μg/ml, and 3 strains of serogroup C with a high level of resistance (MIC, 32 μg/ml). Rifampin-resistant strains were isolated from unvaccinated patients (from 5 to 54 years of age), one of whom died.

A total of 472 strains (472/866 [55%]) were susceptible to penicillin G (Pen^s^) (MIC, ≤0.06 μg/ml), and 388 strains (388/866 [45%]) were penicillin G intermediate (Pen^i^), with an MIC range of 0.094 to 0.25 μg/ml (Table 1). Pen^i^ strains were collected from unvaccinated (184/388 [47%]) and vaccinated (23/388 [6%]) patients through all the age groups. Forty-two percent presented with meningitis, 32% presented with sepsis, 10% presented with meningitis plus sepsis, and the data for the remaining strains were unknown. Eleven percent (44/388) of the patients with Pen^i^ strains died, of which 68% (30/44) had sepsis.

A total of 6 penicillin G-resistant (Pen^r^) strains, with 5 strains in 2006 and 1 strain in 2009, with an MIC range of 0.38 to 0.5 μg/ml (Table 1), were detected. Pen^r^ meningococci were isolated from unvaccinated patients (1 patient with meningitis, 4 patients with sepsis, and 1 patient with unknown clinical presentation), with an age range of 1 to 83 years. The 83-year-old patient, who had sepsis, died.

**FIG 1:**
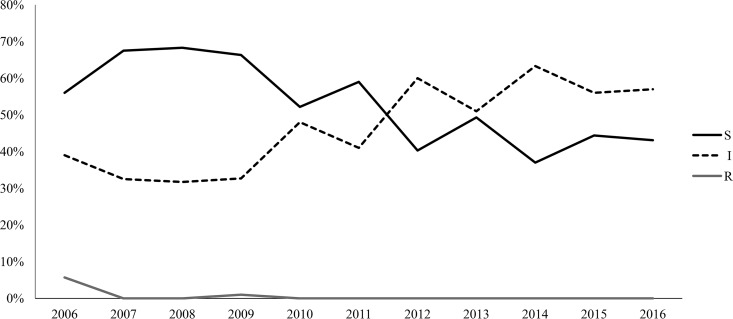
Trend of antimicrobial susceptibility to penicillin G in 866 invasive meningococcal strains, Italy, from 2006 to 2016. S, susceptible; I, intermediate; R, resistant.

As shown in Fig. 1, the antimicrobial susceptibility trend of penicillin G changed over the time frame. In particular, starting from 2012, a statistically significant increase in Pen^i^ strains (*P* < 0.05) has been observed.

The sequence of a 402-bp DNA fragment of the 3′ part of *penA* was obtained for 132 Pen^i^ strains of more recent isolation (2014 to 2016). Twenty-three *penA* alleles were identified, of which *penA248* was the most prevalent. Out of 23 *penA* alleles, 20 alleles coded for a peptide with 5 amino acid substitutions in the C-terminal region of PBP2 (Table 2). *penA327* and *penA648* harbored 4 substitutions (lacking I566V). The *penA1* wild-type allele was found in 3 Pen^i^ strains (MIC, 0.094 to 0.25 μg/ml) (Table 2).

**TABLE 2:** Characterization of 132 Pen^i^ meningococci isolated in Italy, from 2014 to 2016

*penA* allele	No. of strains	No. of substitutions in PBP2	No. of strains^*a*^																		No. of strains by MIC (μg/ml)			
			MenC						MenB										MenY CC23	MenW CC22				
			CC11	CC175	CC22	CC334	CC865	Unknown	CC162	CC167	CC18	CC213	CC269	CC32	CC41/44	CC461	CC865	Unknown			0.094	0.125	0.19	0.25
1	3	0		1				1					1								2			1
7	19	5	19																		5	8	6	
9	9	5					1								1		2	4	1		1	4	3	1
12	1	5																1			1			
13	2	5													1			1				1	1	
14	13	5			1				9				1					1		1	3	7	3	
15	4	5								1					3						3	1		
19	1	5							1														1	
20	15	5																	15		8	3	3	1
25	1	5							1														1	
33	4	5														4							2	2
54	1	5												1								1		
100	1	5												1								1		
144	1	5												1									1	
248	36	5	35					1													9	11	12	4
295	2	5										2									1	1		
327	3	4	3																		2	1		
599	9	5				9															4	4		1
648	1	4													1							1		
685	1	5																		1	1			
773^*b*^	2	5	2																		1	1		
774^*b*^	2	5																2					1	1
775^*b*^	1	5									1										1			
Total	132		59	1	1	9	1	2	11	1	1	2	2	3	6	4	2	9	16	2	42	45	34	11

a MenC, meningococcal of serogroup C; MenB, meningococcal of serogroup B; MenY, meningococcal of serogroup Y; MenW, meningococcal of serogroup W.

b New *penA* allele.

As shown in Table 2, 55% (73/132) of the Pen^i^ strains belonged to serogroup C, of which 81% (59/73) of the strains were associated with clonal complex 11 (CC11); serogroup B, comprising 31% (41/132) of the Pen^i^ strains, was mostly associated with CC162; serogroup Y was associated with CC23 (16/132 [12%]), and serogroup W was associated with CC22 (2/132 [2%]).

Here, in 11 years of IMD surveillance in Italy, invasive meningococcal strains showed a wide range susceptibility to the antimicrobials used for treatment and chemoprophylaxis. The exception was 6 rifampin-resistant strains, of which 3 strains were highly resistant and 2 strains were ciprofloxacin resistant. Of note, an increase in the proportion of Pen^i^ strains, starting from 2012, has been observed.

It is likely that the increase in Pen^i^ strains was due to the spread of the hypervirulent strain C-CC11 that is of a concern in our country (13). The *penA248* allele was the predominant allele and was associated with the finetype C: P1. 5-1, 10-8:F3-6: ST-11 CC11) (data not shown), which is responsible for severe sporadic cases and outbreaks in Italy (13).

Interestingly, 3 Pen^i^ strains harboring the *penA327* allele showed an increased MIC to cefotaxime even though they were within the susceptibility category. Two of these strains were isolated from men who have sex with men (MSM) with sepsis. This occurrence has been already reported by others (4), underlying that the similarity between *penA327* of N. meningitidis and *penA-XXXIV* of N. gonorrhoeae might determine a genetic exchange between the two Neisseria spp. in the urethra (4, 9).

To conclude, resistant meningococci are rare in this country; however, an increase in Pen^i^ strains was observed mainly associated with the spread of C-CC11 meningococci. Because of the concern over the epidemic potential of this strain, it is crucial to link the molecular traits of invasive meningococcal strains with antimicrobial susceptibility, with a particular attention to the emergence of meningococci with reduced susceptibility to cephalosporins (4).
